# Bioinformatics Analysis of the Expression and Prognostic Significance of Transcription Factor YY1 in Gastric Cancer

**DOI:** 10.1002/cnr2.70181

**Published:** 2025-03-15

**Authors:** Wenliang Chen, Huanhuan Wang, Ntiak Achi, Jinjin Hao, Rui Gong, Qiang Zhao

**Affiliations:** ^1^ Department of General Surgery The 2nd Affiliated Hospital of Shanxi Medical University Taiyuan Shanxi China; ^2^ Department of General Surgery Jincheng People's Hospital Jincheng Shanxi China; ^3^ Graduate Department of Shanxi Medical University Taiyuan Shanxi China

**Keywords:** bioinformatics analysis, gastric cancer, immune cell infiltration, Yin Yang 1

## Abstract

**Background:**

Emerging evidence indicates that the transcription factor Yin Yang 1 (YY1) plays a critical role in the carcinogenesis and progression of various human malignancies. YY1 is highly expressed in gastric cancer (GC), raising interest in its role in GC.

**Aims:**

This study aims to analyze the role of YY1 in gastric cancer, investigate its effect on the tumor microenvironment, and assess its potential as a prognostic marker.

**Methods and Results:**

Transcriptomic data and clinical information from GC patients were obtained from the TCGA and UCSC databases. YY1 expression was analyzed using the R “limma” package. Gene ontology (GO) enrichment and Kyoto Encyclopedia of Genes and Genomes (KEGG) pathway analyses were performed with the online tool clusterProfiler. The relationship between YY1 expression levels and the tumor microenvironment was examined in different risk groups of GC patients. Additionally, YY1‐positive staining in 26 clinical GC samples was measured using ImageJ software. Co‐expression analysis was used to identify prognostic genes associated with YY1, and a prognostic risk model was built and optimized.

Results showed that YY1 was significantly overexpressed in 415 GC (*p* < 0.001) and was associated with poorer survival outcomes (*p* = 0.043). GO and KEGG showed that YY1 was involved in key biological processes of the disease. Higher YY1 expression was correlated with lower stromal and immune cell content in the tumor microenvironment. Immunohistochemical staining confirmed YY1 overexpression in GC tissues compared to normal tissues (*p* = 0.0293). Positive correlations were observed between YY1 and the genes MTA1, TTL15, HNRNPU, WDR20, and PPP4R3A. The prognostic model, which included genes significantly associated with YY1 (risk score AUC = 0.690), predicted patient survival better than other clinical variables.

**Conclusion:**

These findings suggest that YY1 plays an important role in the development of GC. Targeting the YY1 pathway may be a potential treatment strategy for GC.

## 1 Background

Gastric cancer (GC) is the fifth most prevalent cancer and the fourth leading cause of cancer‐related mortality globally [1]. Clinically, the prognosis of GC patients is mainly assessed using the TNM staging system and postoperative pathological grading. The TNM system evaluates tumor size and extent (T), lymph node involvement (N), and the presence of distant metastasis (M) [2]. Postoperative pathological grading further evaluates how differentiated and aggressive the tumor is, helping to predict patient outcomes and guide treatment decisions. However, due to the inherent heterogeneity of tumors, even patients with the same TNM stage and pathological grade may have very different survival outcomes. This highlights the complexity of GC and the need for better prognostic biomarkers and personalized treatment strategies to improve patient care and outcomes [3].

Yin Yang 1 (YY1), located on the q32 region of chromosome 14, is a widely expressed zinc finger protein that regulates gene expression [4]. YY1, also known by other names, such as *UCRBP*, *CF1*, *δ*, and *NF‐E*1, is a member of the GLI–Krüppel family of transcription factors characterized by their zinc finger motifs [5, 6]. These motifs allow YY1 to bind DNA and regulate many target genes involved in cell growth, differentiation, and apoptosis [7, 8, 9].

Recent studies have shown that YY1 plays an important role in GC progression. YY1 promotes GC by increasing cell proliferation, invasion, and metastasis. Specifically, it binds directly to the promoter regions of CCDC43 and ADRM1, causing their overexpression and enhancing GC proliferation and metastasis [10]. Additionally, YY1 regulates ATP6V1A, a subunit of vacuolar H+‐ATPase, which contributes to GC invasion and metastasis [11]. YY1 also interacts with SRF and ING5, regulating the transcription of ING5 in GC [12]. Research has also focused on YY1's role in immune regulation. YY1 can help tumor cells escape immune response by regulating LAG‐3 expression in CD8+ T cells within tumors [13]. circ‐ATAD1 promotes GC cell progression by targeting the miR‐140‐3p/YY1/PCIF1 signaling axis [13]. Beyond this, YY1 is also associated with immune cell infiltration in GC. Overexpression of YY1 reduces immune cell infiltration in GC tumors, suggesting a mechanism of immune evasion [14]. Clinically, elevated YY1 expression is associated with poor prognosis in GC patients, emphasizing its potential as a prognostic biomarker [10, 15].

This study aims to investigate the differences in survival among GC patients with similar clinical and pathological characteristics. It also seeks to develop a prognostic risk model based on YY1‐related gene signatures to predict overall survival. Finally, it evaluates YY1 and its associated genes as potential biomarkers and therapeutic targets in GC, offering insights into their roles in GC progression and patient outcomes.

## 2 Materials and Methods

### 2.1 Data Acquisition and Pretreatment

Transcriptome data of GC patients were obtained from the Cancer Genome Atlas (TCGA) database (https://portal.gdc.cancer.gov/, Project Name: Stomach Adenocarcinoma, Project ID: TCGA‐STAD). HTSeq‐Counts data for TCGA‐STAD were downloaded and processed using a Perl script to construct mRNA expression matrices and convert transcriptome data identifiers. Clinical information for these patients was also retrieved from the University of California, Santa Cruz (UCSC) database (https://genome.ucsc.edu/).

In addition, the study cohort included 26 inpatients diagnosed with GC at the Department of General Surgery, the Second Hospital of Shanxi Medical University (Taiyuan, Shanxi, China), between May 2018 and April 2019. All patients underwent surgical treatment, and the diagnosis of GC was confirmed through postoperative pathological examination. None of the patients received radiotherapy, chemotherapy, or other anticancer treatments before surgery. Ethical approval for this study was obtained from the Ethics Committee of the Second Hospital of Shanxi Medical University (no. 2019YX294). Written informed consent was provided by all participants following the principles outlined in the Declaration of Helsinki.

### 2.2 Analysis of YY1 Expression in GC

In the R programming environment (R Studio version 2023.6.2.0, https://posit.co/download/rstudio‐desktop/), the “limma” package was used to process TCGA data and extract the YY1 expression matrix [16]. Differential expression analysis was done with the “ggplot2” and “ggpubr” packages to create plots showing YY1 expression levels in GC. The YY1 expression matrix was then combined with survival data from GC patients. Prognostic survival analysis of the YY1 gene was carried out using R packages like “survival,” “limma,” and “survminer.” Kaplan–Meier survival curves were created with the “ggpubr” package to visualize and analyze the survival outcomes based on YY1 expression levels in GC patients.

### 2.3 Enrichment Analysis of Function and Pathway

Samples were stratified into high and low groups, and differential expression analysis was performed using the “limma” package. Genes were identified as differentially expressed genes (DEGs) based on the criteria of *p* < 0.05, FDR < 0.05, and |logFC| > 1. Enrichment analysis of the DEGs was conducted with the “clusterProfiler,” “org.Hs.eg.db,” “enrichplot,” and “dplyr” packages under thresholds of *p* < 0.05 and FDR < 0.05 [17]. The results were visualized with bubble and bar plots generated by the “ggplot2” package. Gene set enrichment analysis (GSEA) was also carried out using the “limma,” “org.Hs.eg.db,” “clusterProfiler,” and “enrichplot” packages, along with the Molecular Signatures Database (MSigDB) (c2.cp.kegg.v7.4.symbols.gmt data set, https://www.gsea‐msigdb.org/gsea/msigdb/index.jsp). Significant enrichment was defined as *p* < 0.05 and corrected FDR < 0.05. The GSEA results were visualized with the “ggplot2” package to display the enriched pathways graphically.

### 2.4 Analysis of Tumor Microenvironment (TME) and Immune Cell Infiltration

TME scores were calculated from TCGA data using the “limma” and “estimate” packages [18]. Samples were divided into high and low YY1 expression groups, and TME differences were visualized using the “reshape2”, “ggpubr”, and “ggplot2” packages. Statistical significance was represented with asterisks (* for *p* < 0.05, ** for *p* < 0.01, and *** for *p* < 0.001). Gene expression data for 22 immune cell types were obtained, and the CIBERSORT algorithm was applied to the TCGA‐STAD expression matrix data using the “e1071” and “preprocessCore” packages to measure immune cell abundances. This provided detailed information on immune cell infiltration in each sample. Differential analysis of immune infiltration was performed using the “limma,” “vioplot,” and “ggExtra” packages, with a *p* < 0.05 threshold to identify significant differences. Asterisks were used to indicate significance levels. Box plots and correlation graphs showing immune infiltration profiles were created with the “ggplot2” package to clearly display and analyze the immune landscape.

### 2.5 Immunohistochemical Experiments

The two‐step immunohistochemical detection system was used for staining, which included DAB color development, ethanol gradient dehydration, xylene transparency, and sealing with neutral gum. Following these steps, microscopic examination and image capture were conducted. Cells with brown nuclear particles were identified as positive for YY1 expression, and ImageJ software was used to measure YY1‐positive staining. The integrated optical density (IOD) of YY1‐positive staining in GC and adjacent normal tissues was analyzed with GraphPad Prism 8 to compare expression levels.

### 2.6 The Acquisition of Co‐Expressed Genes

Co‐expressed genes associated with YY1 were performed using the “limma” package, with a correlation coefficient threshold (corFilter) set at 0.6 and a significance threshold (pFilter) of 0.001. The “ggExtra,” “ggplot2,” and “ggpubr” packages were subsequently used to visualize these correlations. For each gene pair, correlation coefficients and *p* values were calculated to assess their strength and significance. Scatter plots were created to visually show the relationships between YY1 and its co‐expressed genes, allowing for detailed analysis of their interactions.

### 2.7 Construction and Evaluation of Prognostic Model

Prognosis‐related differential genes were identified using univariate Cox regression analysis with a significance threshold of *p* < 0.05. A LASSO proportional hazards regression model was built using multivariate Cox regression analysis to calculate risk coefficients for the selected genes. The model was optimized to improve prediction accuracy. Using the median risk score as a cutoff, patients were divided into high‐risk and low‐risk groups. Kaplan–Meier survival curves were created with the “Survival” package and the “survminer” package (version 3.8‐3, https://cran.r‐project.org/web/packages/survival/index.html) to compare survival between the two groups. The “pheatmap” package was used to generate risk curves, survival state plots, and heatmaps showing patient risk profiles. Independent prognostic analysis was done with the “survival” package, and a forest plot was made with the “ggplot2” package to show hazard ratios and confidence intervals for each factor. The “survivalROC” package was used to create and show receiver operating characteristic (ROC) curves, which evaluated the model's ability to distinguish risk groups based on gene expression.

### 2.8 Statistical Analysis

Statistical analysis was performed using R software (version 4.3.1) in the R Studio environment (version 2023.6.2.0). A *p* value < 0.05 was used to determine statistical significance for all tests in this study.

## 3 Results

### 3.1 High Expression of YY1 as a Poor Prognostic Factor in GC

**FIGURE 1:**
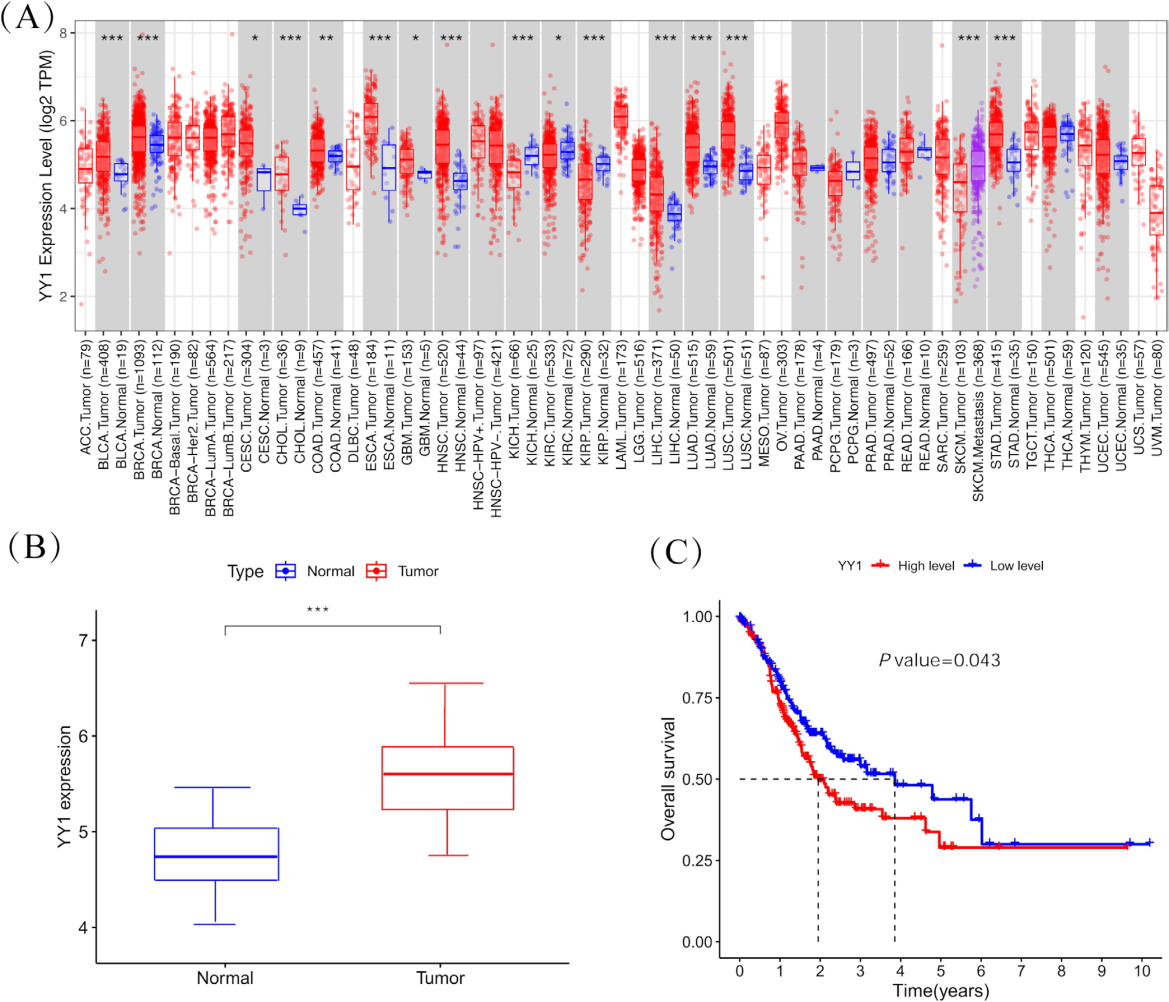
YY1 expression in TCGA gastric cancer. (A) YY1 expression in pan‐carcinoma. (B) The expression level of YY1 is higher in STAD Tumors (*n* = 415) compared to STAD Normal samples (*n* = 35). (C) Prognostic value of YY1 expression in patients with GC. According to the median value of YY1 expression in GC, patients with gastric cancer were stratified into a high‐expression group and a low‐expression group. *p < 0.05; **p < 0.01; ***p < 0.001.

Analysis of the YY1 expression matrix from the TCGA database showed that YY1 expression was significantly higher in GC tissues (*n* = 415) compared to normal tissues (*n* = 35) (*p* < 0.001) (Figure 1A,B). Based on the median YY1 expression value in GC, patients were grouped into high and low expression categories. Kaplan–Meier survival analysis demonstrated a significant association between YY1 expression and patient survival (*p* = 0.043) (Figure 1C). Specifically, patients with low YY1 expression had a much higher 5‐year survival rate than those with high YY1 expression, suggesting that YY1 could be a useful prognostic marker for GC.

### 3.2 Enrichment Analysis of Biological Functions and Pathways

**FIGURE 2:**
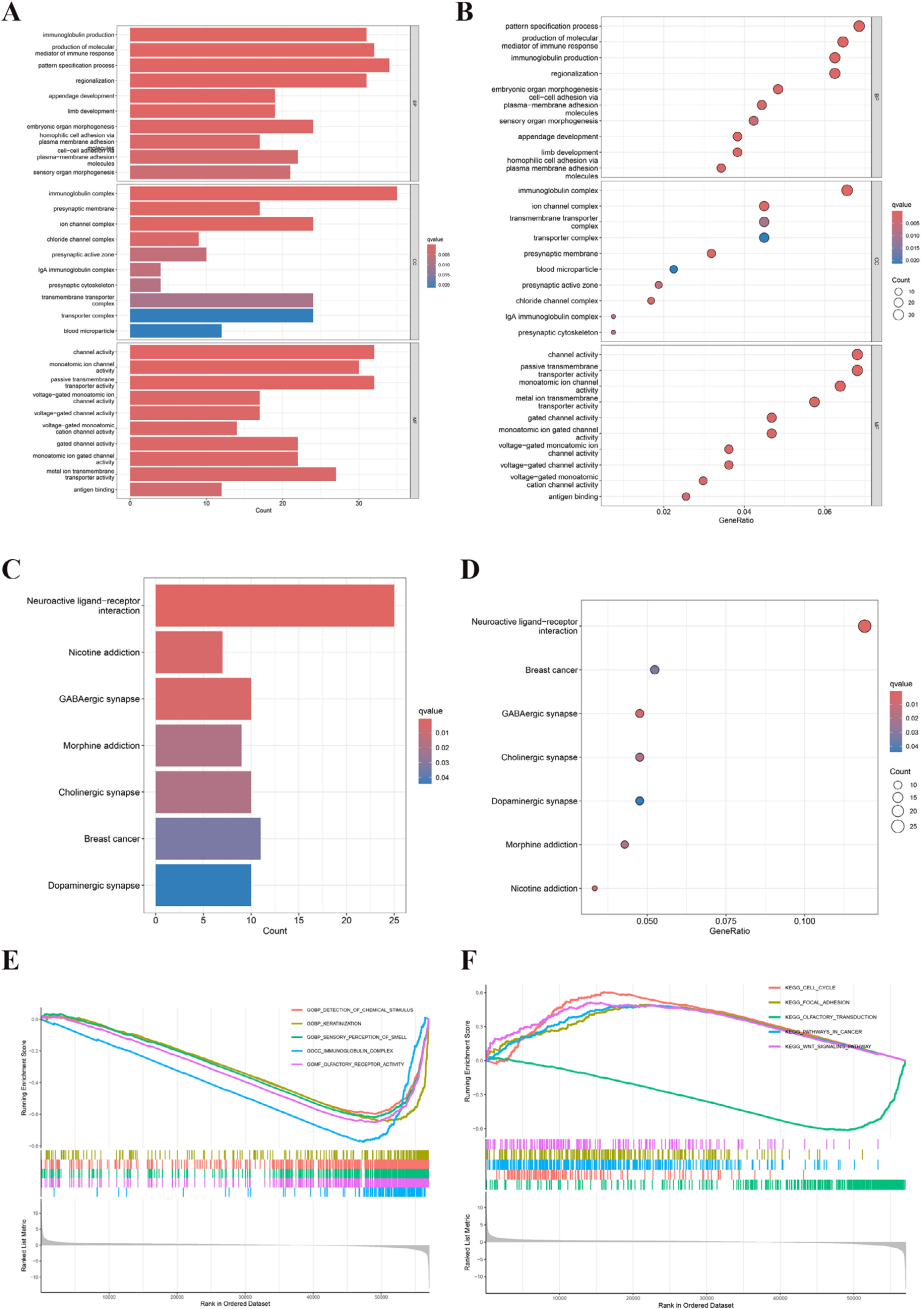
Functions enrichment analysis of YY1. (A) GO enrichment analysis of upregulated DEGs. (B) GO enrichment analysis of downregulated DEGs. (C) KEGG pathway analysis of upregulated DEGs. (D) KEGG pathway analysis of downregulated DEGs. (E) GSEA enrichment analysis of GOs. (F) GSEA enrichment analysis of KEGG pathways.

To explore the biological functions and pathways associated with YY1 in GC, enrichment analyses were performed. Gene ontology enrichment analysis showed that YY1 was related to pathways involving the immunoglobulin complex, ion channel complex, immune response mediator production, channel activity, and passive transmembrane transporter activity among DEGs (Figure 2A,B). Similarly, the Kyoto Encyclopedia of Genes and Genome analysis indicated pathways such as cholinergic synapse, GABAergic synapse, and dopaminergic synapse (Figure 2C,D).

To refine the analysis further, GSEA was performed using upregulated and downregulated DEGs. The results showed that pathways enriched in downregulated DEGs were mainly related to olfactory transduction, while upregulated DEGs were linked to the cell cycle, focal adhesion, and Wnt signaling pathway (Figure 2E,F). High expression of these DEGs was associated with processes like ferric iron binding, chylomicron assembly, and neutrophil‐mediated transduction. In contrast, low expression of these DEGs was related to Type I interferon receptor binding, chemical stimuli detection, and olfactory receptor activity. These findings highlight the important roles of YY1 in GC, suggesting it is involved in key biological processes and pathways that may influence disease progression and guide treatment strategies.

### 3.3 TME and Immune Cell Infiltration Analysis

**FIGURE 3:**
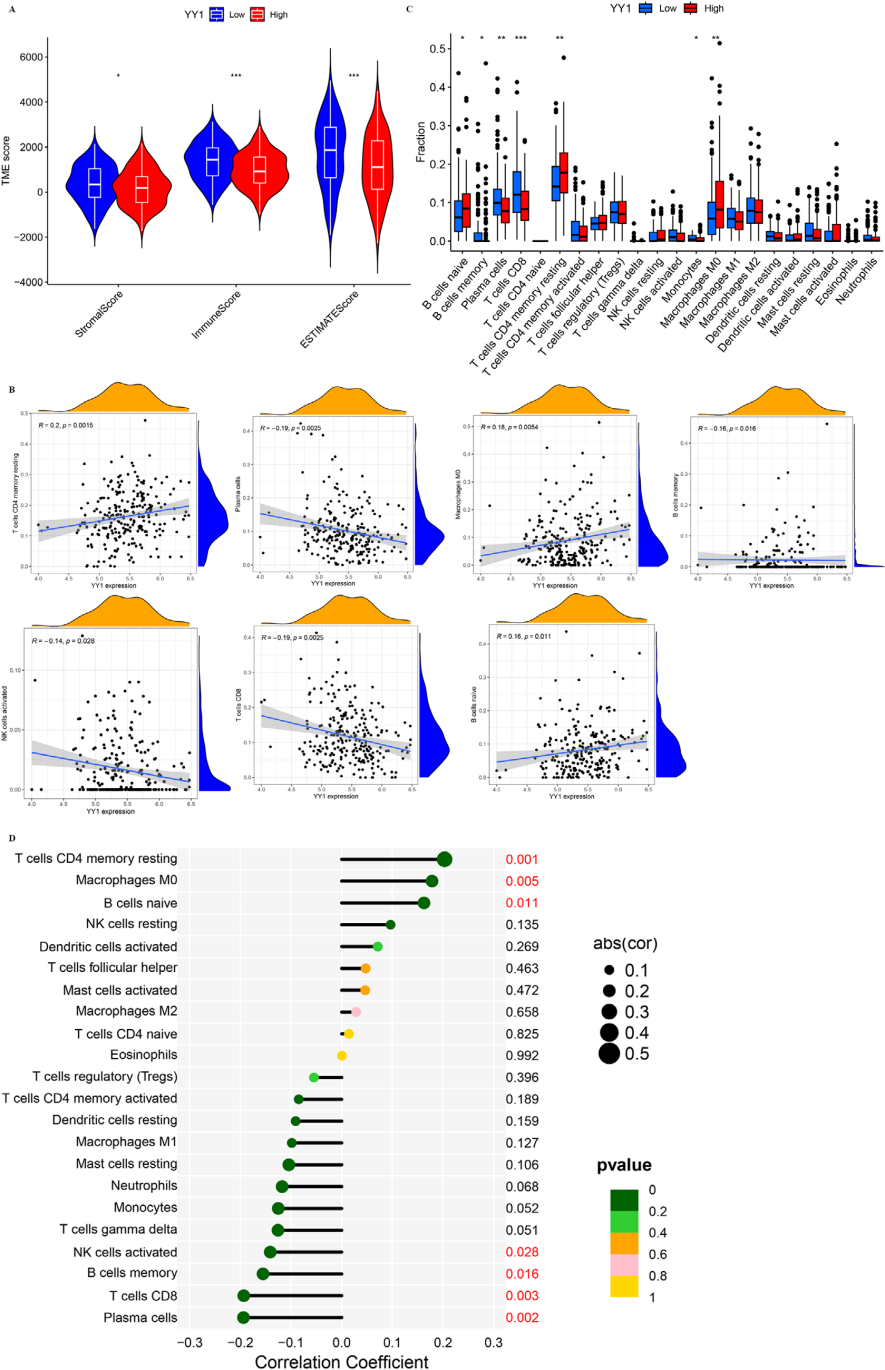
Analysis of tumor microenvironment and immune cell infiltration among patients in different risk groups. (A) Tumor microenvironment score in two groups. (B) Correlation analysis of immune cell infiltration and YY1 expression level. (C) Differential analysis of immune cell infiltration in two groups. (D) Correlation analysis of immune cell population and YY1 expression level. *p < 0.05; **p < 0.01; ***p < 0.001.

YY1 expression levels were divided into high and low groups based on the median value. Significant differences were found in stromal score (*p* < 0.05), immune infiltration score (*p* < 0.001), and ESTIMATE score (*p* < 0.001) between these groups (Figure 3A). These scores were higher in tissues with low YY1 expression, indicating that higher YY1 expression is linked to lower stromal cell and immune cell content within the TME (Figure 3B). Moreover, the analysis showed clear differences in immune cell profiles between the GC and normal tissue samples (Figure 3C). Correlation analysis between YY1 expression and specific immune cell types revealed significant negative correlations with natural killer (NK) cells (*r* = −0.140963, *p* = 0.028344), memory B cells (*r* = −0.155232, *p* = 0.015648), plasma cells (*r* = − 0.193884, *p* = 0.002490), and CD8+ T cells (*r* = − 0.193292, *p* = 0.002529) (Figure 3D). In contrast, YY1 expression was positively correlated with naive B cells (*r* = 0.162505, *p* = 0.011353), memory CD4+ T cells (*r* = 0.203071, *p* = 0.001494), and macrophage M0 (*r* = 0.178314, *p* = 0.005405) (Figure 3D). These results suggest that YY1 expression may affect the immune cell composition in the GC TME, potentially influencing disease progression and immune response.

### 3.4 YY1 Protein Expression in GC Tissue

**FIGURE 4:**
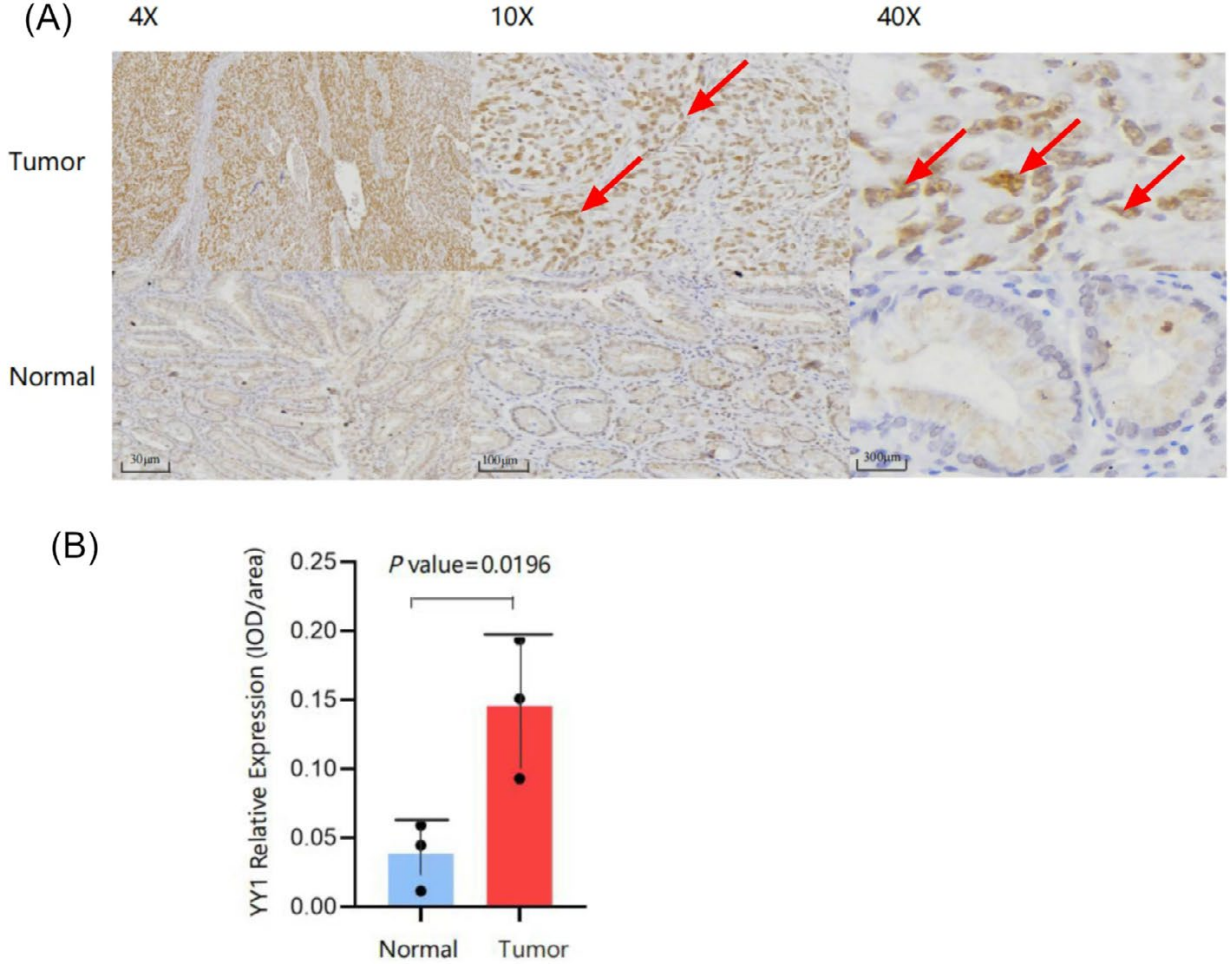
Immunohistochemistry staining of YY1 in GC tissues. (A) Positive expression of YY1 in the nucleus (red arrow). 4×,10×, and 40× representing the magnification of the objective lens. (B) The immunostaining score of YY1 in gastric cancer and adjacent normal tissues.

Immunohistochemical staining showed that YY1 expression was mainly localized in the nucleus, with some presence in the cytoplasm. Imaging was done using Motic DSAssistant software at 4×, 10×, and 40× magnifications. The areas with YY1‐positive cells in GC and normal tissues were quantified using ImageJ software (Figure 4A). Semi‐quantitative staining scores showed significantly higher YY1‐positive staining in GC tissues compared to normal tissues (*p* = 0.0293) (Figure 4B). These results suggest that YY1 protein overexpression is specific to GC tissue, supporting its potential as a biomarker for GC pathogenesis and diagnosis.

### 3.5 Co‐Expression Analysis

**FIGURE 5:**
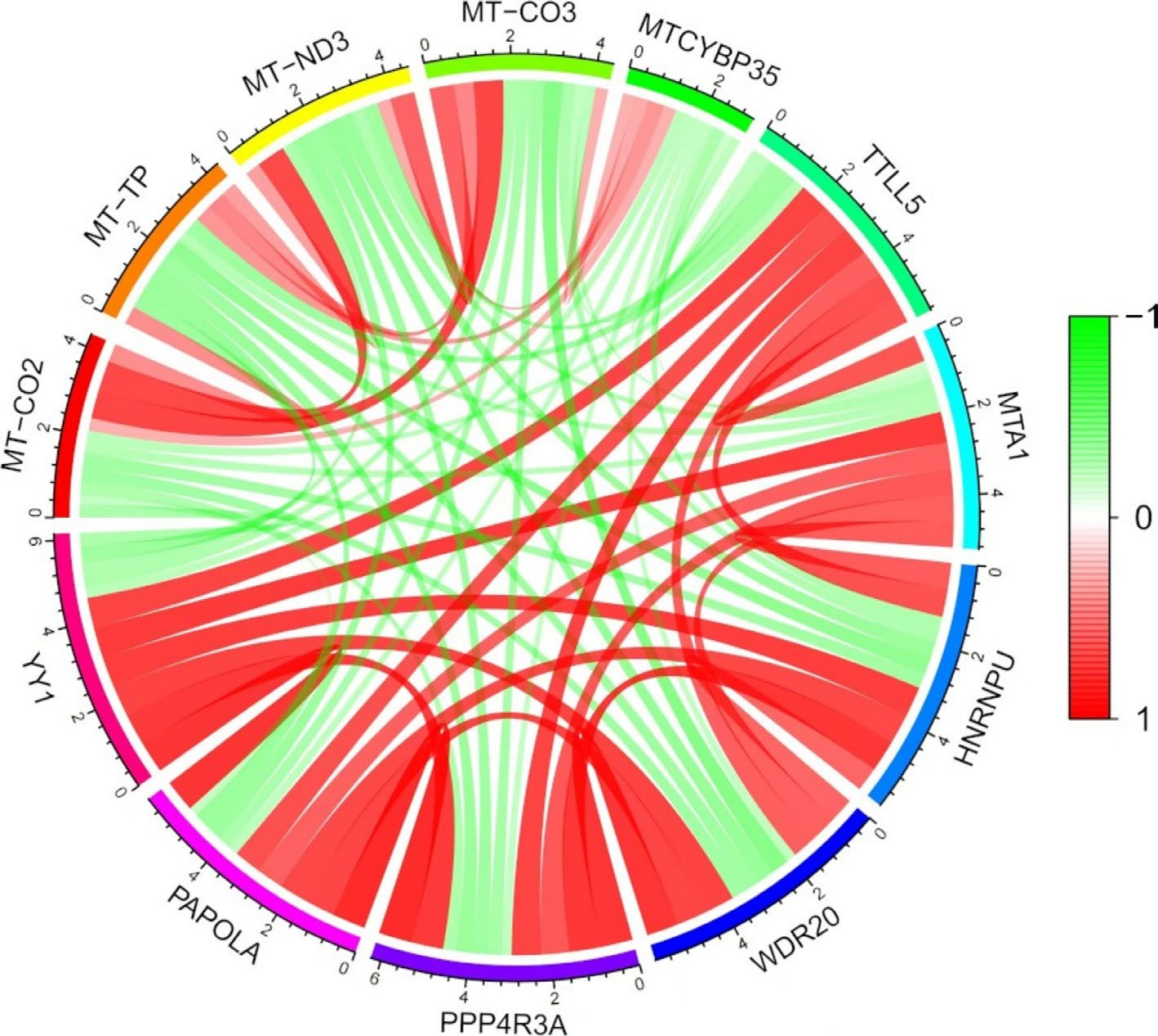
Co‐expression analysis of YY1 with other related genes.

Co‐expression analysis showed positive correlations between YY1 and genes such as TTL15, MTA1, HNRNPU, WDR20, and PPP4R3A. On the other hand, negative correlations were found between YY1 and genes like MT‐CO2, MT‐TP, MT‐ND3, MT‐CO3, and MTCYBP35 (Figure 5). These results suggest that YY1 may interact with and regulate these genes in GC. These associations offer useful insights into the role of YY1 and its involvement in disease progression.

### 3.6 Construction and Evaluation of the Prognostic Model

To create a strong prognostic model, univariate and multivariate Cox regression analyses were carried out. The model was defined by the following formula:

Risk score=−0.42535×ALG11+0.910403×KBTBD2−0.37175×POLR1A−0.4042×PPROSER1−0.37019×RRBM15+0.023839×YY1

**FIGURE 6:**
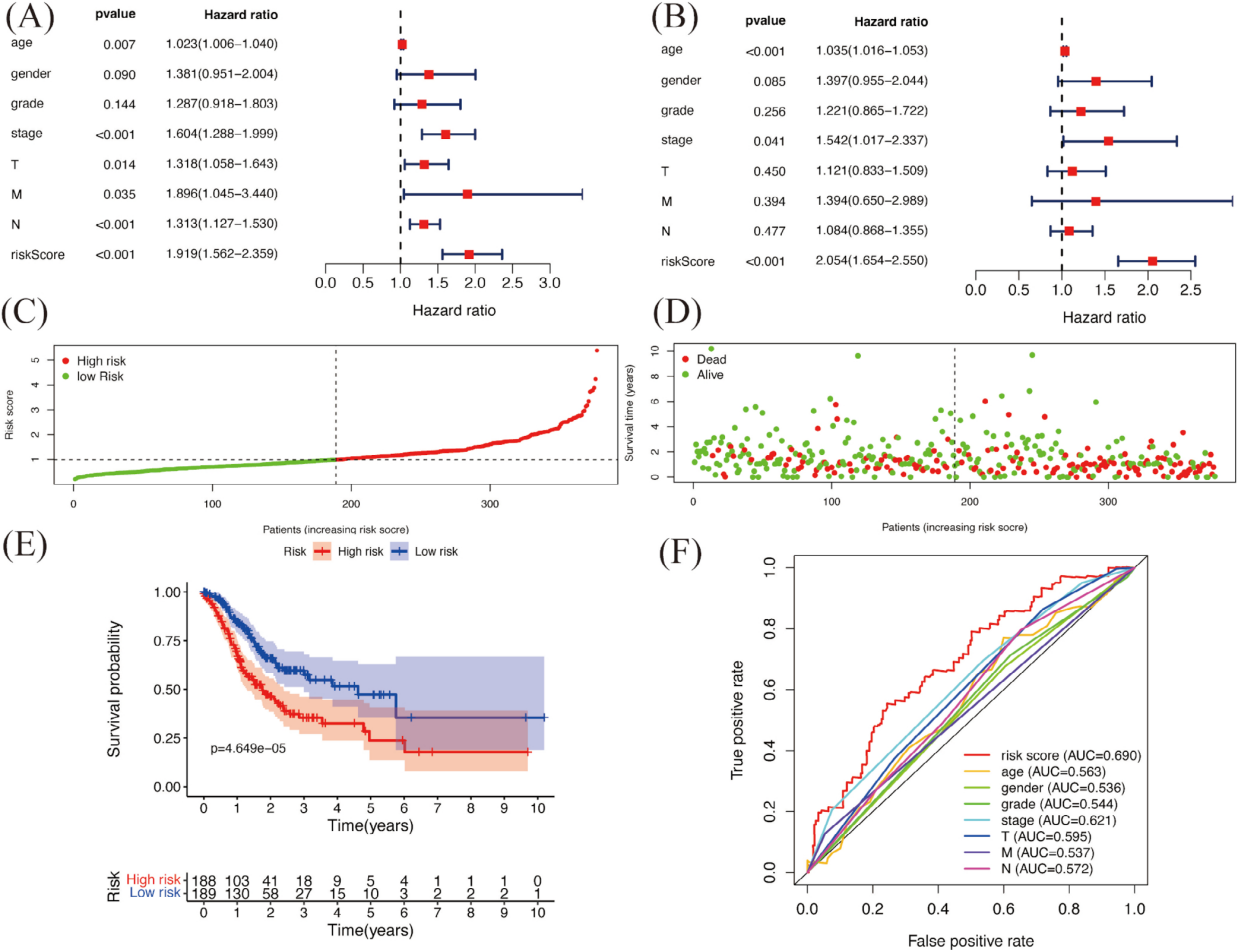
Construction and evaluation of the prognostic model. (A) Univariate independent prognostic factor analysis. (B) Multivariate independent prognostic factor analysis. (C) Prognostic model risk curve. (D) Patients' risk score with survival time. (E) Kaplan–Meier survival curve. (F) ROC curve for validating the prognostic model.

Univariate analysis found several factors linked to survival, such as age, clinical stage, TNM classification, and risk score (Figure 6A). Multivariate analysis confirmed that age, clinical stage, and risk score were independent predictors of prognosis (Figure 6B), with *p* values of age and clinical stage less than 0.001. These results show that the model is effective for predicting patient outcomes.

Based on the risk score, patients were divided into high‐risk and low‐risk groups using the median score (Figure 6C,D). Kaplan–Meier survival analysis showed a significant difference in survival between the two groups (*p* = 4.649e−05; Figure 6E). The overall survival rates for the whole group were 67.5% at 1 year, 35.4% at 3 years, and 23.7% at 5 years. In contrast, the low‐risk group had better survival rates: 84.4% at 1 year, 57.4% at 3 years, and 47.3% at 5 years. The results confirm that lower risk scores are linked to longer survival, supporting the model's prognostic value.

To check the model's accuracy, we performed ROC curve analysis. The area under the curve (AUC) for the risk score was 0.690, higher than other clinical variables, showing its better predictive power (Figure 6F). These findings highlight the model's potential to help guide treatment for GC patients.

## 4 Discussion

GC is characterized by significant molecular and phenotypic heterogeneity [19, 20], with tumor tissues consisting of diverse clonal subpopulations, each of which exhibits distinct phenotypes and genetic profiles. This intratumoral molecular diversity makes it harder to understand the disease and its treatment. Emerging evidence suggests that such heterogeneity contributes to the limited efficacy of chemotherapy and immunotherapy, as well as the poor prognosis commonly associated with GC [21, 22]. Identifying distinct patient subpopulations with prognostic and/or predictive biomarkers is therefore important for creating targeted treatments and improving precision oncology.

YY1 is a multifunctional protein that acts as a transcription factor, RNA‐binding protein, and chromatin regulator. It plays a key role in processes like tumor cell growth, invasion, epithelial–mesenchymal transition, DNA damage response, and carcinogenesis [23]. Our bioinformatics analysis has revealed that YY1 is highly expressed in GC tissues, which was confirmed by immunohistochemistry. Elevated YY1 expression correlates with poorer prognosis in GC patients, making it a potential prognostic biomarker [24]. Further analysis indicated that YY1 overexpression activates key pathways, such as the cell cycle and Wnt signaling, both of which are involved in carcinogenesis. This may help explain the poor prognosis associated with YY1 overexpression. Other evidence suggests that silencing YY1 in GC cells inhibits multiple important signaling pathways, including JNK/MAPK, ER, Wnt/β‐catenin, ERK/MAPK, and HIF‐1α [25]. Furthermore, the GSEA results also show how YY1 promotes GC proliferation and metastasis by regulating the CCDC43‐ADRM1 axis [10].

CD8+ T cell‐mediated cellular immunity plays a critical role in eliminating tumor cells, and the effectiveness of immunotherapy largely depends on the activation of these cells. However, cancer cells can avoid immune surveillance by suppressing CD8+ T cell activity, enabling their survival through immune escape mechanisms [26, 27]. In analyzing the TME and immune cell infiltration, we showed a complicated relationship between YY1 expression and the immune landscape in GC. Specifically, YY1 expression negatively correlated with immune cell populations such as memory B cells, NK cells, CD8+ T cells, and plasma cells. High YY1 expression was associated with reduced content of both the extracellular matrix and immune cells, suggesting an immunosuppressive effect. Notably, high YY1 expression in tumor tissues was often linked to a decrease in immune cell numbers, potentially contributing to immune evasion and poor prognosis [28, 29]. Merenstein et al. further elucidated a potential molecular mechanism underlying this phenomenon. Their findings revealed that YY1 overexpression regulates the transcription of LAG‐3 in CD8+ T cells, leading to their inactivation and depletion. This loss of CD8+ T cell functionality facilitates immune escape and supports tumor progression [13]. Interestingly, YY1 expression was positively correlated with naive B cells, memory CD4+ T cells, and M0 macrophages, suggesting a more complicated role of YY1 in modulating the immune microenvironment. In some cases, CD4+ T cell subtypes and M0 macrophages can promote tumor growth, which needs further study [30, 31, 32].

Co‐expression analysis revealed interesting relationships between YY1 and other genes in GC. Specifically, YY1 was positively correlated with TTL15, MTA1, HNRNPU, WDR20, and PPP4R3A, which may suggest potential functional interactions or shared regulatory mechanisms. A particularly significant finding was the positive correlation between YY1 and MTA1, a gene linked to metastasis. This suggests that YY1 may contribute to tumor progression and metastasis [33]. Furthermore, YY1 expression was negatively correlated with mitochondrial genes such as MT‐CO2, MT‐TP, MT‐ND3, MT‐CO3, and MTCYBP35. This suggests that YY1 may linked to mitochondrial function or energy metabolism in cancer cells, providing new insights into the metabolic mechanisms behind YY1's in GC [34].

The development of a six‐gene prognostic model that includes YY1 expression represents a significant advancement in the risk stratification of GC patients. The model effectively distinguishes between high‐risk and low‐risk groups with different survival outcomes, showing its potential for clinical treatment. Compared to traditional clinical variables, this model exhibits better predictive accuracy, as shown by its higher AUC values. Multivariate analyses also confirm the independent prognostic value of the risk scores, with age and cancer stage as additional factors, highlighting the strength of our model. By combining molecular biomarkers with clinical parameters, this approach supports the growing focus on personalized cancer treatment.

A literature search using keywords like YY1, risk scores, prognostic models, multivariate analysis, and GC did not find studies that include all these elements together. This study appears to be the first to combine a risk score based on YY1 expression with clinical parameters like age and GC stage in a prognostic model. These results have important clinical implications. Since YY1 is overexpressed and linked to poor clinical outcomes, strategies to inhibit YY1 activity or expression could offer promising treatments. Potential approaches might include small molecule inhibitors, antisense oligonucleotides, or other innovative therapies [35, 36]. Furthermore, the immunosuppressive effect linked to high YY1 expression suggests that targeting YY1 could enhance the efficacy of immunotherapy. Combining YY1 suppression with immune checkpoint inhibitors or other immunotherapeutic strategies may yield synergistic benefits [37, 38].

### 4.1 Limitations

This study primarily focuses on the role of YY1 in GC tumorigenesis through bioinformatics approaches. While many genes and pathways are linked to YY1 overexpression, the specific ones driving GC cell growth and invasion are still unknown. Future studies using in vitro and in vivo models are needed to clarify these mechanisms. Also, the effect of YY1 on immune cell infiltration in the GC microenvironment requires further study. Research shows that gene sets regulated by YY1 are mainly linked to intestinal‐type GC and some cases of diffuse‐type GC, highlighting the need to study these two subtypes further [21].

## 5 Conclusion

In this study, we created a new prognostic risk model that combines YY1 expression with clinical variables like age and tumor stage to predict clinical outcomes in GC. Our results show that YY1 overexpression is closely related to key aspects of tumor biology, such as immune evasion, metabolic weaknesses, and pathway‐specific alterations. What makes our research new is the combination of YY1 expression, risk scores, and multivariate analysis in the context of GC, which has not been done before. These results lay the groundwork for further studies on YY1 as a prognostic marker and a therapeutic target in GC, possibly leading to better outcomes for this disease.

## Author Contributions

**Wenliang Chen:** conceptualization (lead), data curation (equal), formal analysis (equal), funding acquisition (lead), investigation (lead), methodology (lead), project administration (lead), resources (lead), software (equal), supervision (lead), validation (lead), visualization (equal), writing – original draft (lead), writing – review and editing (equal). **Huanhuan Wang:** conceptualization (supporting), data curation (equal), formal analysis (equal), methodology (lead), resources (equal), software (lead), validation (lead), writing – original draft (equal), writing – review and editing (equal). **Ntiak Achi:** data curation (equal), formal analysis (supporting), methodology (supporting), resources (equal), validation (equal), visualization (supporting), writing – original draft (equal), writing – review and editing (equal). **Jinjin Hao:** data curation (supporting), formal analysis (equal), software (supporting), visualization (equal), writing – original draft (equal), writing – review and editing (equal). **Qiang Zhao:** investigation (equal), methodology (supporting), resources (supporting), software (equal), writing – original draft (equal), writing – review and editing (equal). **Rui Gong:** data curation (supporting), resources (supporting), software (equal), validation (equal), writing – original draft (equal), writing – review and editing (equal).

## Ethics Statement

This study followed the Declaration of Helsinki and was approved by the Ethics Committee of the Second Hospital of Shanxi Medical University (approval no. 2019YX294).

## Conflicts of Interest

The authors declare no conflicts of interest.

## Data Availability

The data that support the findings of this study are available in the UCSC database at (https://genome.ucsc.edu/).
